# Assessing the potential for specimen pooling to streamline nosocomial surveillance of methicillin-resistant *Staphylococcus aureus* (MRSA)

**DOI:** 10.1128/spectrum.02585-25

**Published:** 2025-11-14

**Authors:** Isabella Pagotto, Mohammed Alqahtani, Bryn Joy, Gregory R. McCracken, Ian R. Davis, Jason J. LeBlanc, Glenn Patriquin

**Affiliations:** 1 Department of Microbiology and Immunology, Dalhousie University 152982, Halifax, Nova Scotia, Canada; 2 Division of Microbiology, Department of Pathology and Laboratory Medicine, Nova Scotia Health 432234 , Halifax, Nova Scotia, Canada; 3 Microbiology Section, Pathology and Medical Laboratories Department and Blood Banks, Security Forces Hospital 48061 , Riyadh, Saudi Arabia; 4 Medical Sciences Program, Faculty of Sciences, Dalhousie University 3688, Halifax, Nova Scotia, Canada; 5 Department of Pathology, Dalhousie University 152985, Halifax, Nova Scotia, Canada; 6 Department of Medicine, Dalhousie University 152981, Halifax, Nova Scotia, Canada; Montefiore Medical Center and Albert Einstein College of Medicine, Bronx, New York, USA

**Keywords:** MRSA, surveillance, PCR, pooling, cost, antibiotic resistance

## Abstract

In hospitals, identification of methicillin-resistant *Staphylococcus aureus* (MRSA) is important to reduce possible transmissions and serious outcomes. Traditional culture and susceptibility testing requires 48–72 h, whereas Xpert MRSA polymerase chain reaction (PCR) can provide accurate MRSA detection in <1 h. Unfortunately, the high cost of such commercial PCRs precludes their use in many laboratories. Using MRSA as a model, this study hypothesized that specimen pooling in a setting of low prevalence could reduce PCR costs and provide rapid results. A total of 424 sequential nasal/groin specimens submitted for MRSA detection were subjected to routine culture-based detection using chromogenic media, and suspect colonies were confirmed using mass spectrometry and cefoxitin disk diffusion testing. These specimens were also pooled 1:8 or processed individually by Xpert MRSA PCR. Analytical sensitivity of PCR with and without pooling was compared to culture using triplicate 10-fold serial dilutions of an MRSA reference strain. The analytical sensitivity and clinical performance of specimen pooling paired with Xpert MRSA PCR were equivalent to traditional culture-based detection. Of specimen pools, 66.0% (35/53) were MRSA negative, and 34.0% (18/53) were MRSA positive. Pool resolution by PCR showed similar results as culture, identifying 116 MRSA-negative and 28 MRSA-positive specimens. At a prevalence of 6.6% (28/424), 1:8 specimen pooling with Xpert PCR provided equivalent results to culture-based methods and reduced the overall number of PCR reactions by 53.5%. Compared to individual PCR testing, specimen pooling would lower overall PCR costs, but the feasibility of this approach and the extent of benefits afforded would depend on MRSA prevalence.

**IMPORTANCE:**

Identifying antibiotic-resistant bacteria like methicillin-resistant *Staphylococcus aureus* (MRSA) is important to prevent their spread and potentially life-threatening infections. MRSA can be detected using bacterial culture and antibiotic susceptibility testing but requires up to 3 days for results. Molecular detection methods like polymerase chain reaction (PCR) are more rapid (<1 h), but their high cost prevents implementation for many laboratories. To reduce PCR costs, specimen pooling was considered. With specimen pooling, swabs from multiple individuals are combined and tested together. If pools are negative, all their members are considered negative. If pools are positive, each swab is tested individually to identify the one(s) with MRSA. By reducing the number of PCRs required, pooling reduces PCR costs. In this study, 6.7% of samples were MRSA positive, and pooling reduced overall PCR costs by 54%, provided results in 1–2 h, and identified the same number of MRSA cases as the comparator (i.e., culture).

## INTRODUCTION

Antibiotic-resistant organisms (AROs) such as methicillin-resistant *Staphylococcus aureus* (MRSA), vancomycin-resistant *Enterococcus* (VRE), and carbapenem-resistant organisms (CROs) are increasing problems for healthcare systems worldwide (1–3). AROs are resistant to commonly used antibiotics for treatments of infections, and the lack of appropriate treatment options contributes to significant morbidity and mortality (3, 4). In hospitals, infection prevention and control (IPAC) programs are vigilant to identify AROs and reduce possible transmission routes by implementing mitigation strategies such as reemphasizing good hand hygiene practices, isolating colonized or infected patients, utilizing personal protective equipment, and restricting visitor access (5, 6). Common IPAC approaches for monitoring and preventing ARO transmissions include targeted surveillance methods like contact tracing after the discovery of known cases or prospective surveillance by testing large cohorts of people (e.g., all patients of a hospital unit) to identify sporadic cases that might have gone unnoticed through targeted surveillance (5, 6).

Clinical microbiology laboratories support ARO identification using a variety of methods. Traditional methods for MRSA detection use a combination of antibiotic susceptibility testing following culture and identification of *S. aureus* through methods like agglutination reactions or matrix-assisted laser desorption/ionization time-of-flight (MALDI-ToF) (7–10). For large batch testing from prospective surveillance samples, selective chromogenic media can be used to facilitate identification of MRSA at a reasonable cost but with a turnaround time of 24–72 h when paired with the necessary confirmatory culture and susceptibility testing (7, 8). Molecular methods in real time, such as polymerase chain reaction (PCR), can be applied directly on clinical specimens to detect *S. aureus* and identify MRSA, and results can be provided in less than an hour with some assays like the Cepheid Xpert MRSA assay (9–11). The main disadvantage of molecular tests is their high cost, which often precludes their use for routine testing and surveillance in many laboratories. Given PCR is often more sensitive than culture-based methods, this project assessed the use of specimen pooling paired with PCR testing for MRSA as a strategy to lower the overall cost of PCR testing, reduce human resources required in the microbiology laboratory, and gain efficiencies in terms of speed of result reporting to help guide IPAC control strategies.

**Fig 1:**
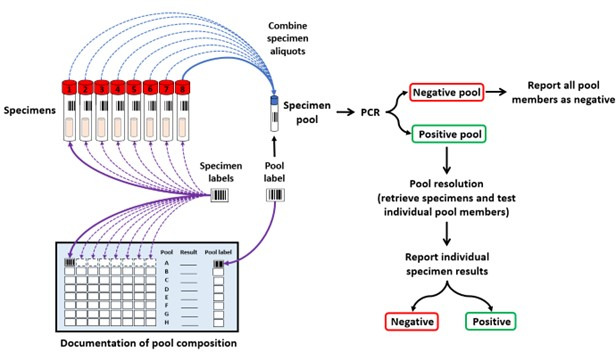
The principle of specimen pooling. In the diagram, a pool depth of 1:8 is shown, where eight specimens are combined into a single tube for simultaneous testing using PCR (blue arrows). If the pool PCR result was negative, all pool members were considered negative. If a pool PCR result was positive, the pool was resolved by retesting each specimen individually to identify the positive specimen(s). Specimen traceability can be maintained with documentation of specimen barcodes, either manually on laboratory worksheets (purple arrows) or electronically through the hospital laboratory information system.

Specimen pooling (i.e., group testing) combines specimens from different individuals for simultaneous testing in a single reaction. When specimen pools are negative, all specimens within the pool are considered negative, and rapid results are achieved at the cost of a single specimen reaction. Only positive pools require subsequent individual specimen testing to identify origin(s) of the positive result(s) (Fig. 1). Specimen pooling was originally described by Dorfman in 1943 (12) and continues to be used for the detection of bloodborne pathogens (e.g., human immunodeficiency virus, hepatitis C virus, and hepatitis B virus) by the Canadian Blood Services (www.blood.ca), the American Red Cross (13), and for diagnostic testing of other infectious diseases (e.g., *Chlamydia trachomatis*, *Mycobacterium tuberculosis*, and malaria) (14–17). More recently, specimen pooling was widely used during the COVID-19 pandemic when resources were limited, SARS-CoV-2 prevalence was low, and testing demands were high (18–21). Pool depths below 1:10 are often used to minimize the risk of generating false-negative results while maintaining the benefits of group testing. Pooling efficiencies gained are dependent on both pool depth and the prevalence of disease, as an increase in pooled specimens per assay would require increased resolution assays in high disease prevalence (18–21). For settings of low disease prevalence as seen with ARO surveillance in Canada, few pools would require resolution, thereby increasing the speed of results, reducing laboratory cost of PCR testing, and streamlining laboratory workflow. Low specimen pool depths and testing on a simple PCR instrument (Xpert PCR) have been explored for surveillance of CROs and VRE with some successes (22, 23). However, except for pooling swabs collected for MRSA detection from different anatomical sites from the same individual (24–26), the benefits of specimen pooling from different individuals had not yet been explored for MRSA surveillance.

To streamline microbiological testing and IPAC investigations, this study assessed whether specimens from multiple individuals could be pooled and tested simultaneously for MRSA using PCR in a setting of low prevalence. The feasibility and performance of specimen pooling was assessed at pool depths of 1:8, and MRSA PCR was evaluated with or without pooling compared to routine culture and sensitivity methods used for MRSA detection in the Canadian province of Nova Scotia.

## MATERIALS AND METHODS

### Estimated impact of pool depth and prevalence on pooling efficiency and testing costs

**Fig 2:**
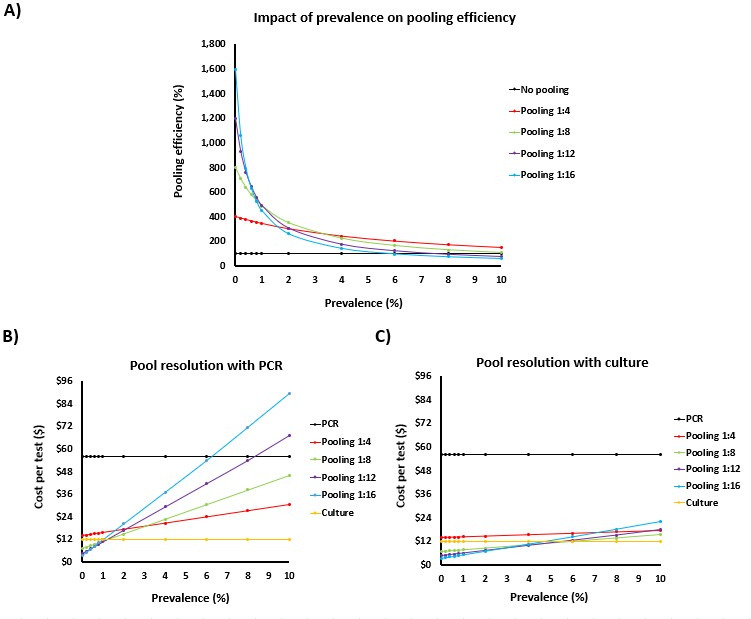
Impact of prevalence and pool depth on testing efficiency. (**A**) Pooling efficiency was modeled based on specimen pooling or not. For cost analysis, testing cost for each pool depth and prevalence were considered for different testing strategies: (**B**) specimen pooling with PCR testing along with positive pool resolution with culture and (**C**) specimen pooling with PCR testing along with PCR to resolve positive pools. Results are shown for bacterial culture (yellow), PCR without pooling (black), and PCR with specimen pooling at depths of 1:4 (red), 1:8 (green), 1:12 (purple), and 1:16 (blue).

To illustrate the potential benefits of specimen pooling, pooling efficiency was modeled at a prevalence ranging from 0% to 10% using no pooling or pool depths of 1:4, 1:8, 1:12, and 1:16 (Fig. 2). These pool depths were considered due to the ease of processing multiples of 4 (congruent with sample capacities of PCR instruments available in Nova Scotia). Higher pool depths were not considered, given concerns of potentially generating false-negative results. For each % prevalence, an arbitrary number of 1,000 specimens was used to determine the number of PCRs required for each pool depth, the number of positive and negative pools, and downstream impacts like pooling efficiency and costs. Overall, pooling efficiency (%) was calculated as follows: {[total number of specimens ÷ (1/pool depth)] ÷ [(number of PCRs required) ÷ (1/pool depth)]} × 100. The resulting pooling efficiencies with PCR are presented in Fig. 2A.

The number of PCRs required for specimen pooling for each pool depth, as well as the number of positive and negative pools, was also used for cost analyzes. Modeling test costs (without human resources) was undertaken for each pool depth and prevalence while considering different testing strategies: (i) specimen pooling with PCR testing along with positive pool resolution with culture (Fig. 2B); and (ii) specimen pooling with PCR testing along with PCR to resolve positive pools (Fig. 2C). For both strategies, the total number of cultures and PCRs required was calculated from the estimated number of positive and negative pools generated for each pool depth and prevalence, and a cost of $56 was applied per PCR, $4.86 per negative culture, and $5.10 for positive cultures with MRSA confirmation, based on local costing. Total costs per strategy, pool depth, and prevalence were each divided by the number of specimens to generate the cost per test.

### Bacterial culture, *S. aureus* identification, and susceptibility testing for MRSA

All culture and susceptibility testing followed routine practices in the Division of Microbiology, Department of Pathology and Laboratory Medicine, Nova Scotia Health (Halifax, NS). Following routine nose/groin sampling using an eSwab in liquid Amies media (Copan Diagnostics Inc., Murrieta, CA), each specimen was transported to the laboratory and stored at 4°C until testing. For bacterial culture, a 30 µL inoculum was streaked onto Denim Blue Chromogenic MRSA Screening Agar (Oxoid Company, Nepean, ON) using a Walk Away Specimen Processor (bioMérieux, St-Laurent, QC) to ensure standardization, followed by incubation of the plates aerobically at 37°C for 16 h. Colonies suspicious for MRSA were those that were blue and pinpoint to 1 mm in diameter, whereas white colonies represented methicillin-resistant coagulase-negative *Staphylococcus* and were ignored. Blue colonies were subjected to Gram staining and to MALDI-ToF detection using a BD Bruker MALDI Biotyper (Becton Dickinson Canada Inc., Mississauga, ON) to confirm the identification of *S. aureus*. MRSA was confirmed using disk diffusion testing with a 30 µg cefoxitin disk (Becton Dickinson and Company, Sparks, MD) on Oxoid Mueller–Hinton agar as per Clinical Laboratory Standards Institute guidelines (M100 Performance Standards for Antimicrobial Susceptibility Testing, 35th Edition).

### MRSA PCR

For molecular detection of MRSA, the Xpert MRSA NxG kit (Cepheid, Sunnyvale, CA) was performed as per manufacturer instructions. The Xpert MRSA NxG assay uses primers and probes targeting the *mec* genes (i.e., *mecA* and *mecC*, methicillin/oxacillin resistance genes) in one fluorescent channel, and detection of the *orfX*-SCC*mec* junction specific to *S. aureus* occurs in the second fluorescent channel. Results are expressed with threshold cycle (Ct) values for each channel target, and overall results are interpreted by the manufacturer software.

### Specimen pooling

For specimen pooling, 100 µL liquid Amies from eight primary specimens were combined into a single microtube (i.e., pool depth 1:8) manually using a micropipette. For large batches, a VOYAGER 8-Channel Adjustable Tip Spacing Pipette (INTEGRA Biosciences Corp., Hudson, NH) was used to prepare multiple pools simultaneously. Of the specimen pooled mixture, 300 µL was used for Xpert MRSA PCR, consistent with the volume used for individual specimens. If the pool PCR result was negative, all pool members were considered negative. If a pool PCR result was positive, the pool was resolved by retesting each specimen individually to identify the positive specimen(s). A summary of the process is described in Fig. 1. Of note, specimen traceability was maintained with documentation of specimen barcodes both manually on laboratory worksheets and electronically through the hospital laboratory information system using specimen pooling software developed by Cerner Millenium. Specimens were held at 4°C in organized racks until PCR was complete. This allowed for rapid retrieval and resolution of positive pools. Long-term storage of specimens occurred at −80°C.

### Method comparisons

**Fig 3:**
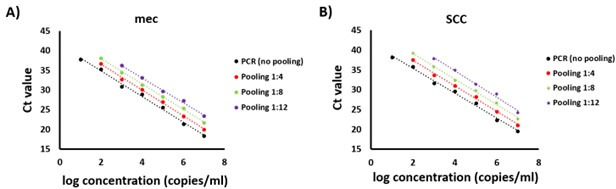
Analytical sensitivity comparisons of MRSA PCR testing with and without specimen pooling. Tenfold serial dilution of an MRSA isolate was performed in three independent experiments and subjected to MRSA PCR testing. Bacterial dilutions representing pool depths of 1:4 (red), 1:8 (green), and 1:12 (purple) were compared to those processed without pooling (black). Panels **A** and **B** represent Ct values obtained for the *mec* (*mecA* and *mecC* genes) and SCC targets of MRSA, respectively.

Two sets of experiments were used to compare MRSA detection using traditional culture-based methods or PCR with or without pooling. First, analytical sensitivity for each method was assessed using 10-fold serial dilutions of *S. aureus* American Type Culture Collection 33591 (i.e., an MRSA reference strain). All dilutions and pooling were performed in MRSA PCR-negative specimens collected in eSwab media. For quantification, each 10-fold serial dilution was plated onto the Denim Blue chromogenic/selective agar to determine colony-forming units (CFUs) per milliliter. Ct values for *mec* and SCC targets obtained with MRSA PCR in the absence of pooling or with pooling at depths of 1:4, 1:8, and 1:12 were plotted against the concentration of each bacterial dilution to estimate the limits of detection of each approach. PCR results were expressed as log genome copy equivalents (copies) per milliliter and represented the average values from three independent experiments (*n* = 3) (Fig. 3).

Next, prospective testing was performed to compare clinical performance using 424 clinical specimens submitted for routine MRSA surveillance from hospital wards to the Division of Microbiology, Nova Scotia Health. Specimens spanned a 14-day period from 15 to 29 June 2023. To more accurately estimate MRSA prevalence, duplicate swabs collected from individuals testing positive within 90 days were excluded as per local testing protocols. Testing was performed using culture-based methods as per routine standard of care and compared to results obtained with PCR without pooling or those obtained with specimen pooling at a depth of 1:8. Sequential specimens were divided into batches of eight for specimen pooling at a depth of 1:8, and Xpert MRSA PCR was performed within 24 h from receipt in the laboratory. Ct values for both *mec* and SCC were plotted to compare values obtained for PCR with and without pooling.

## RESULTS

### Impact of prevalence and specimen pooling on testing efficiency and costs

Predictive modeling looking at pooling efficiency suggested that PCR would be greatest at low prevalence and at higher pool depths (Fig. 2A). For example, at an MRSA prevalence of 0%, pools depths of 1:4, 1:8, 1:12, and 1:16 would generate pooling efficiencies with PCR of 400%, 800%, 1,200%, and 1,600%, respectively, compared to testing without pooling used as the comparator and considered 100%. As MRSA prevalence increases, efficiencies decline from the greater numbers of positive PCR pools that require resolution. With a pool depth of 1:4, pooling efficiency declines to 150% at an MRSA prevalence of 10%, while the same prevalence and a pool depth of 1:8 show negligible gains with a pooling efficiency of 108%. With the higher pool depths of 1:12 and 1:16, pooling efficiencies are lost at an even lower prevalence: 8% and 6%, respectively (Fig. 2A).

MRSA prevalence would also have an impact on testing cost, as higher prevalence would trigger more positive pools that would require resolution. However, the extent of cost incurred would be dependent on the strategy used for pool resolution following specimen pooling and PCR testing (Fig. 2B and C). Using culture-based methods for pool resolution would be the most cost-sparing at any of the modeled pool depths or prevalence, but the speed of results afforded by PCR testing and pooling would only be seen for specimens in negative pools. Positive or negative specimens that are part of positive pools would take 48–72 h for resolution. Using PCR testing on pools and for pool resolution would achieve the fastest results, but cost would increase with MRSA prevalence (Fig. 1B). At a prevalence of 6% and 8%, specimen pooling at depths of 1:16 and 1:12 would become more expensive than PCR on individual specimens without prior pooling. Specimen pooling at depths of 1:4 and 1:8 would be cost-effective compared to PCR alone even at a prevalence of 10%, although the cost would increase with prevalence and would far exceed those of bacterial culture. A third strategy was proposed whereby PCR pools would be resolved using PCR, but individual specimens would concomitantly be tested by culture-based methods (data not shown). This method would be the most prudent, given that some MRSA strains may contain variants of *mec* or SCC target regions that may not be detected using Xpert MRSA PCR (27). While this may circumvent the possibility of false-negative PCRs and provide rapid preliminary results for IPAC patient management, the cost of this approach would be a combination of a PCR-based pooling strategy with PCR-based positive pool resolution, as well as an addition of the cost of bacterial culture.

### Impact of specimen pooling on analytical sensitivity

Using 10-fold serial dilutions of an MRSA reference strain, PCR without pooling was shown to be the most sensitive. The analytical sensitivity of specimen pooling at depths of 1:4 and 1:8 is equivalent to culture at approximately 100 copies/mL, which represented approximately 1 CFU using culture onto Denim Blue agar. The pool depth of 1:12 was the least sensitive, being approximately 10-fold less sensitive than pool depths of 1:4 and 1:8 and approximately 100-fold less sensitive than non-pooled PCR. Overall, with each multiple for pool depth, Ct values of either the *mec* or SCC PCR targets shifted by approximately 1.5.

### Impact of specimen pooling at a depth of 1:8 on the diagnostic detection of MRSA

Traditional bacterial culture was compared to PCR testing with or without pooling at a depth of 1:8 using 424 clinical specimens submitted from hospital wards for routine MRSA surveillance. Specimen pooling paired with PCR testing was able to identify all MRSAs identified using bacterial culture, and no additional MRSAs were detected using PCR alone. It should be noted that PCR was able to identify MRSA in specimens containing as low as 1 CFU. With specimen pooling, there was no increase in failure due to PCR inhibitors, as monitored using the internal control from the Xpert PCR.

## DISCUSSION

Commercial kits for molecular detection of MRSA (e.g., PCR) offer the promise of a rapid and reliable result (9–11) but the disadvantage of high cost, as compared to traditional culture-based methods. In this investigation, pooling specimens at a ratio of 1:8 followed by Xpert PCR reduced the number of PCR reactions required while maintaining equivalent results to culture-based methods. The modeling data in this study suggested that PCR paired with pooling will not achieve cost neutrality with culture-based methods but can be cost-sparing when compared with individual PCR in low-prevalence settings.

Pooled PCR testing for MRSA in clinical settings offers several advantages, including faster turnaround times, cost-effectiveness, and reduced laboratory workload. This approach is particularly beneficial in settings with low MRSA prevalence, where most pools are expected to be negative. The potential to streamline laboratory operations and improve the efficiency of diagnostic workflows without sacrificing accuracy compared with culture-based methods may be particularly important in clinical environments where timely results are essential for IPAC practices. Faster turnaround times can directly impact patient management decisions, enabling quicker isolation measures and potentially reducing the overall spread of MRSA within healthcare facilities.

The commercial PCR assay used in this investigation (e.g., Xpert MRSA NxG) has been shown to be sensitive and specific for MRSA detection and have high negative predictive value (11); however, pooling specimens had not been previously evaluated for MRSA detection. Pooling is relatively easy to implement, as seen widely in its application to PCR during the COVID-19 pandemic (18–21). However, pooling is not without limitations. Specimen pools were tested on nose/groin swabs in liquid Amies, but the Xpert MRSA has only been licensed for use with nasal swabs. This might limit the generalizability of the results. Specimen pooling also adds an element of complexity to testing and comes at a cost of reduced sensitivity, particularly at high pool depths. Despite the reduced analytical sensitivity with pooling in this study, no impact was noted on clinical sensitivity during the clinical validation compared to traditional culture-based detection. Another concern with manual pooling is the potential risk of specimen contamination, yet this could be mitigated in part by the use of automation and good laboratory practices. Another real-world challenge in implementation is that specimens often do not arrive in desirable multiples for pooling. This study was performed in a 14-day period, and testing was performed when sufficient specimen pools were reached. It is likely that prevalence would vary over time and between different institutions, as would the number of specimens submitted to hospitals throughout the day. The number of specimens in a given time can complicate the pooling process and may require adjustments in workflow to accommodate these, and the cost benefits of pooling would be reduced with lower pool depths.

An important limitation is that this study was conducted at a single center, focusing on a single organism and a single PCR test, which reduces the generalizability of the results. Additionally, when considering different settings, the feasibility of adopting specimen pooling with PCR depends on various factors, such as MRSA prevalence, available laboratory resources, and the need for rapid result reporting. In this setting, MRSA prevalence was low compared to other regions, but in jurisdictions with MRSA rates lower than in the current study, the combined pooling and PCR method may be more advantageous. In this study, Xpert MRSA PCR was used due to its simplicity, high sensitivity, and availability in many laboratories across Nova Scotia, but this PCR comes at a high cost. It is possible that other commercial assays might provide better cost-effectiveness with specimen pooling, such as a highly sensitive low-cost PCR that might achieve higher pool depths and faster throughput. However, PCRs can vary in performance (28–30) and would need to be thoroughly evaluated even if the cost was sufficiently low. Future studies should explore the application of specimen pooling with other PCR methods considering prevalence, test performance, and costs.

This study provides proof of concept and discussion points around specimen pooling for MRSA; however, MRSA is not the only ARO of interest in hospital settings. Keidar-Friedman et al. (22) recently used specimen pooling for rectal swabs submitted for Xpert PCR detection of VRE and CRO. Their results showed that the performance of rectal swab pooling at a depth of 1:6 was adequate for the detection of some CROs, but this strategy did not perform well for VRE, showing many false-positive and false-negative results. Specimen pooling 1:5 and testing for CROs using Xpert PCR was supported in a previous study by Zhang et al. (23). As previously discussed for MRSA (28–30), the results obtained in this study on MRSA and previous studies on other AROs should not be extrapolated to other molecular detection methods or settings. Furthermore, assuming test performance using specimen pooling was shown to be adequate following validation, it could potentially reduce the turnaround times for MRSA, VRE, and CRO detection, which in turn would significantly influence IPAC strategies, potentially leading to more efficient use of hospital resources, timely isolation of infected patients, and optimized use of antibiotics. Factors such as bed and room assignment, patient flow, personal protective equipment usage, and antibiotic use should be considered to determine if the overall impact justifies the costs. Understanding the broader implications of specimen pooling on hospital operations, resource utilization, and patient care is important if it is to be successfully implemented.

Our study highlights the potential of specimen pooling paired with Xpert MRSA PCR as a strategy for MRSA detection in low-prevalence settings. While the results are promising, further research is needed to explore the potential applications and impacts of specimen pooling using Xpert or other molecular methods for the detection of AROs, with a careful consideration for prevalence, test performance, and overall costs.
